# Molecular Mechanism of Metformin Regulating the Regeneration of Planarian *Dugesia japonica* Through *miR-27b*

**DOI:** 10.3390/ijms26157092

**Published:** 2025-07-23

**Authors:** Kexin Yang, Minmin Feng, Chunmei Zhang, Zelong Zhao, Dandan Yin, Linxia Song, Zhenbiao Xu

**Affiliations:** School of Life Sciences and Medicine, Shandong University of Technology, Zibo 255000, China; 19811718781@163.com (K.Y.); minamin1997@126.com (M.F.); 18632842808@163.com (C.Z.); zelong0222@gmail.com (Z.Z.); YinDandansdut@163.com (D.Y.); slxch@163.com (L.S.)

**Keywords:** metformin, *Dugesia japonica*, *DjmiR-27b*, *DjPax6*, regeneration

## Abstract

Metformin is one of the most commonly used medications to treat type 2 diabetes. In addition to lowering blood sugar, it can also promote the regeneration of certain organs or tissues. Planarian *Dugesia japonica*, with its remarkable regenerative capacity, has become an important model organism for studying pharmacology and regenerative medicine. Planarian eyespot regeneration involves precise tissue regeneration via mechanisms like cell proliferation, differentiation, and gene regulation following body damage. Experiments on planarian eyespot regeneration have confirmed that 1 mM metformin significantly promotes regeneration. Through analysis of the regenerating planarian miRNA database and the metformin-treated transcriptome database, combined with target gene prediction by TargetScan, the *DjmiR-27b/DjPax6* axis was finally determined as the research focus. qPCR showed that metformin significantly affects the expression levels of *DjmiR-27b* and *DjPax6*. *DjPax6* was identified as the target gene of *DjmiR-27b* through dual luciferase reporter gene analysis. Functional experiments revealed that metformin regulates the expression of *DjPax6* via *DjmiR-27b*, thereby influencing the regeneration of planarian eyespots. In situ hybridization showed that both *DjmiR-27b* and *DjPax6* are expressed throughout the entire body. This study reveals the molecular mechanism of metformin regulating planarian regeneration through miRNA, providing further insights into its role in the field of regeneration.

## 1. Introduction

Metformin is a kind of biguanide drug used in the treatment of type 2 diabetes, which plays a role mainly by activating AMP-activated protein kinase (AMPK). Metformin, a biguanide-class drug and first-line therapeutic for type 2 diabetes mellitus (T2DM), exerts its primary pharmacologic action through activation of AMP-activated protein kinase (AMPK). This activation leads to inhibition of hepatic gluconeogenesis, suppression of glucagon secretion, and enhancement of insulin sensitivity, collectively contributing to effectively promoting blood glucose reduction [1,2,3]. In addition to lowering blood sugar, metformin also has many functions, such as delaying the aging process and extending lifespan in both mice and nematodes [4,5,6]. Moreover, it has been found to reduce the risk of Alzheimer’s disease in diabetics [7]. Metformin can also improve obesity-induced inflammation in mice with a high-fat diet [8]. In addition, it has shown the ability to inhibit the growth and metastasis of various types of tumor cells, including breast cancer, liver cancer, and gastric cancer, breast, hepatic, and gastric carcinomas [9,10]. Furthermore, metformin promotes myelin regeneration in the central nervous system. In neural systems, metformin enhances central nervous system (CNS) remyelination and restores the regenerative ability of aged oligodendrocyte progenitor cells in rats [11]. It also facilitates heart regeneration in zebrafish by triggering autophagy [12]. In an ischemia–reperfusion (I/R) model in mice, metformin was shown to attenuate cardiac and hypertrophic remodeling after reperfusion [13].

Planarian *Dugesia japonica* (*D. japonica*) has a strong regenerative ability and possesses extraordinary regenerative capacity, serving as a key model organism for developmental biology, pharmacology, and regeneration research [14,15,16,17,18]. The regenerative ability of planarians is mainly attributed to the abundant neoblasts in their body, which constitute approximately 20–30% of the total number of cells in the planarian [19,20]. Neoblasts have the ability of self-renewal, proliferation, differentiation, and migration. self-renewal, proliferative capacity, multipotent differentiation, and migratory activity. They can differentiate into cells of any tissue type, thereby providing a cellular basis for the regeneration of planarian individuals [19,20]. The regeneration process of planarians establishes polarity along the anterior–posterior and dorsal–ventral axes, with the anterior fragment regenerating a head and the posterior fragment regenerating a tail after amputation; the dorsal and ventral sides can be correctly distinguished and form specific tissues and structures [14]. Planarian regeneration is related to a variety of signaling pathways, such as Wnt and EGFR [21]. Research has shown that the Wnt signaling pathway can affect antero-posterior govern anteroposterior patterning and the proliferation of stem cells during planarian regeneration [22,23]. The inhibition of *Evi/Wls* and *Wnt1* genes can lead to ectopic phenomena in head formation, and the suppression of the *Notum* gene can result in head loss or the formation of double tails of the regenerating planarian [24,25]. The EGFR pathway is involved in the mediation of differentiation of various tissues such as the intestine, pharynx, and photoreceptors of planarian [26]. There are also some genes involved in regulating the regeneration process of planarians, and abnormal expression of these genes can affect their regeneration. Knockdown of the *Djfoxk1* gene can inhibit regeneration of the cranial ganglia, resulting in the failure of eye regeneration [27], while the degradation of the *Djequinox* gene results in abnormal regeneration of planarian buds [28]. Therefore, planarian regeneration is a complex network of regulatory processes orchestrated regulatory network influenced by various genes and microenvironments.

MicroRNA (miRNA) is an approximately 20 nt noncoding single-stranded RNA endogenous noncoding RNA molecule encoded by endogenous genes. It regulates many important biological processes, including cell proliferation, differentiation, migration, apoptosis, and tissue/organ homeostasis [29,30]. MiRNAs exert their biological functions mainly by binding to the 3′UTR 3′ untranslated (3′UTR) region and leading to the degradation and inhibition of expression of their target genes [29,30]. Further research found that miRNA can also bind to the CDS region or 5′UTR 5′ untranslated (5′UTR) region of their target genes [31,32]. MiRNA-regulated target genes also include tRNA, rRNA, and lncRNA (long noncoding RNA) in addition to mRNA [31,32]. Research on miRNAs in regeneration has found that *miR-203* hinders zebrafish fin regeneration by suppressing the Wnt signaling mediator *lef1* [33]; the *miR-183* cluster (*miR-96*, *miR-182*, and *miR-183*) plays a role in regulating hair cell regeneration in zebrafish ears [34]. In planarians, the *miR-124* family is linked to brain and visual system regeneration [35], and *miR-8b* participates in brain and eye regeneration [36]. These findings show miRNAs’ regulatory roles in regeneration biology.

Metformin can modulate the expression of various miRNAs, thereby helping to achieve its biological functions. The *let-7* microRNA family possesses tumor-suppressive activity and tumor suppressor functions. Recent studies have shown that metformin significantly increases the levels of *let-7a* in cancer stem cells in mouse xenograft models, antagonizing cancer progression, inhibiting tumorigenesis [37]. Metformin can reduce insulin resistance in human adipocytes by downregulating the expression of *miR-223* and improving the IRS/Akt/GLUT4 signaling pathway [38]. In BALB/c wild-type mice, metformin enhances the antitumor activity of NK cells via overexpression of *miR-150* and *miR-155* [39]. Other studies have revealed that the biological actions of metformin are mediated through the downregulation of genes specific to cancer stem cells (CSCs) and the re-expression of miRNAs (*let-7a*, *let-7b*, *miR-26a*, *miR-101*, *miR-200b*, and *miR-200c*) that are typically lost in pancreatic cancer, indicating that metformin may help overcome therapeutic resistance in pancreatic cancer cells [40].

*MiR-27* is a functionally diverse miRNA family; it is divided into two subtypes: *miR-27a* and *miR-27b* in humans. *MiR-27b* is closely related to bone metabolism and tumor development. The decrease in the expression level of *miR-27b* can increase the expression of its target gene *MMOL/LP-13*, leading to a decrease in the expression of *COLII collagen type II (COL2A1),* and causing intervertebral disc degeneration [41]. In the field of cancer research, it has been found that high expression of *miR-27b* can inhibit the expression of *PPARγ*, promote the proliferation and invasion of cervical cancer cells, and increase the risk of cervical cancer [42]. Low expression of *miR-27b* may increase the occurrence and development of gastric cancer by targeting *GSPT1* or *ROR1* [43,44]. *Pax6* is a transcription factor essential for the development of tissues like eyes, central nervous system, and endocrine glands in vertebrates (e.g., mice, zebrafish) and invertebrates (e.g., fruit flies, nematodes) [45]. Studies in animal models (e.g., mice, frogs, chickens) showed it regulates eye development and orchestrates retinogenesis by controlling cell proliferation, differentiation, and survival in eye tissues [46]. Mutations or deletions of the *Pax6* gene are linked to congenital eye malformations and neurological developmental abnormalities, such as anophthalmia, microphthalmia, iris defects, iridohypoplasia, and brain/retina retino-cortical dysplasia [46,47,48]. It has been reported that *miR-27b* is expressed in the human retinal pigment epithelial cell line ARPE-19 [49]. In the serum and retinas of 5–10-week-old diabetic mice, *miR-27b-3p* is one of the significantly dysregulated miRNAs. These miRNAs can regulate the expression of *VEGF*, *CREB1*, *BDNF*, and *PPAR-α* in diabetic retinas and may serve as potential biomarkers and therapeutic targets for early diabetic retinopathy [50]. In early retinal development, the expression of *Pax6* is regulated by multiple signaling pathways, including BMP and TGF-β, although its exact mechanisms of action remain unclear [51].

In this study, the function of *DjmiR-27b* and *DjPax6* in planarian eyespot regeneration regulated by metformin was investigated. It was found that metformin can regulate planarian eyespot regeneration by modulating the expression of the target gene *DjPax6* through *DjmiR-27b*. This study provides a theoretical basis and mechanistic insight for the research of metformin and miRNA in the field of regeneration.

## 2. Results

### 2.1. Effect of Metformin on Eyespot Regeneration in Planarians

**Figure 1:**
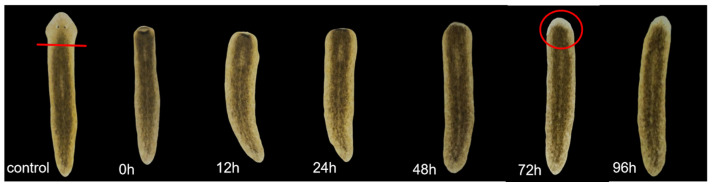
Regeneration process of eyespots in planarians. The red line represents the cutting site, and the red circle indicates that the planarian has completed its eyespot regeneration.

**Figure 2:**
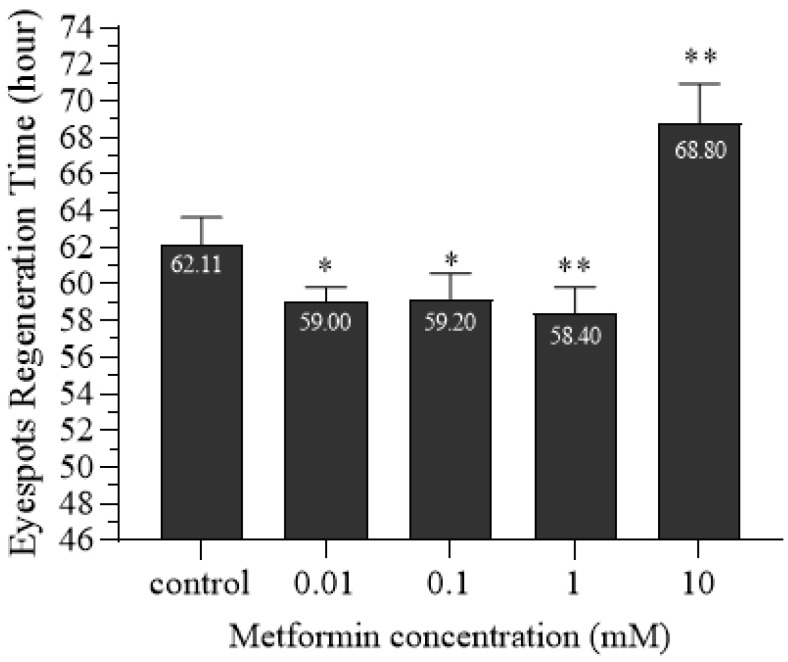
Time of eyespot regeneration in planarians treated by metformin (* *p* < 0.05; ** *p* < 0.01).

Previous studies have shown that low concentrations of metformin can promote planarian’s eyespot regeneration, with 1 mM demonstrating the most significant promoting effect [52]. In this study, we used 0.01 mM, 0.1 mM, 1 mM, and 10 mM metformin solutions for verification. Planarians were transversely cut posterior to the auricles and cultured in clean water and the above concentration of metformin solution. The process of eyespot regeneration was observed, and the time of regeneration was recorded. Planarians cultured in clean water were used as controls. Through preliminary exploration, we found that the regeneration time of planarian eyespots is between 48 and 72 h (Figure 1). The time for planarian eyespot regeneration in the control group was 62.11 h, while the 0.01 mM, 0.1 mM, 1 mM, and 10 mM metformin-treated groups were 59.00 h, 59.20 h, 58.40 h, and 68.80 h, respectively. Compared to the control group, 0.01 mM, 0.1 mM, and 1 mM metformin significantly promoted eyespot regeneration, with 1 mM metformin exhibiting the strongest promoting effect. In contrast, 10 mM metformin significantly inhibited regeneration (Figure 2).

### 2.2. Effect of Metformin on the Expression of DjmiR-27b and Its Target Gene DjPax6 in Regenerating Planarians

TargetScan was used to predict the target genes of *DjmiR-27b*, and the results are shown in Appendix A, Table A1. By analyzing the regenerating planarian miRNA database [53] and the metformin-treated planarian transcriptome database [52] of our laboratory, we hypothesized that *DjPax6* might be related to planarian eyespot regeneration, and *DjPax6* was selected for further study. Sequence homology using NCBI Blast revealed that it shares 98% homology with the *Pax-6B* gene of *D. japonica*.

**Figure 3:**
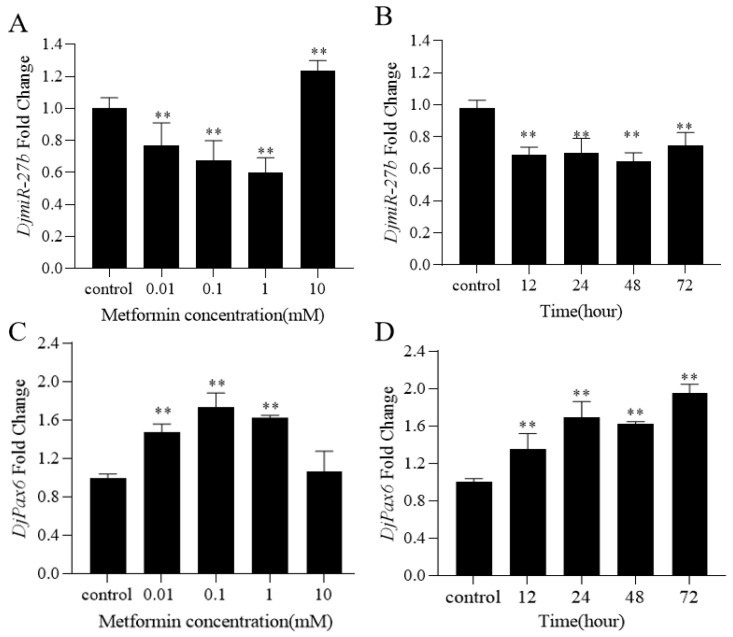
Expression changes in *DjmiR-27b* and *DjPax6*. Expression changes in *DjmiR-27b* (**A**) and *DjPax6* (**C**) treated with different concentrations of metformin for 48 h, and the expression changes in *DjmiR-27b* (**B**) and *DjPax6* (**D**) at different time points treated with 1 mM metformin in the regenerating planarians (** *p* < 0.01).

Planarians with a body length of 0.8–1 cm were selected, and their eyespots were removed to obtain the regenerating planarians, which were then cultured in water and metformin solutions of 0.01 mM, 0.1 mM, 1 mM, and 10 mM, respectively, for 48 h. Then, qPCR was used to detect the expression levels of *DjmiR-27b* and *DjPax6*. Planarians cultured in clean water were used as controls. The results are shown in Figure 3A,C. Compared to the control group, the expression levels of *DjmiR-27b* in 0.01 mM, 0.1 mM, and 1 mM metformin groups were significantly downregulated, and that of the *DjPax6* gene was significantly upregulated. In the 10 mM metformin group, the expression level of *DjmiR-27b* was significantly upregulated, and there was no significant change in *DjPax6* expression level. This indicates that metformin of low concentration can significantly suppress the expression level of *DjmiR-27b* and promote the expression level of *DjPax6* in the regenerating planarians.

To investigate the effects of different durations of metformin treatment on the expression of *DjmiR-27b* and *DjPax6* in regenerating planarians, planarians were treated with 1 mM metformin for 12 h, 24 h, 48 h, and 72 h, respectively, with those cultured in clean water as controls. Then qPCR was used to detect the expression of *DjmiR-27b* and *DjPax6*. The results showed that the expression level of *DjmiR-27b* in the regenerating planarians treated with 1 mM metformin was significantly downregulated at these time points, with a trend of first increasing, then decreasing, and then increasing again (Figure 3B). The expression level of *DjPax6* was significantly upregulated, with the same trend as that of *DjmiR-27b* (Figure 3D). This indicates that 1 mM metformin treatment for different durations can significantly suppress the expression level of *DjmiR-27b* and promote that of *DjPax6* in the regenerating planarians.

### 2.3. Correlative Expression of DjmiR-27b and DjPax6

**Figure 4:**
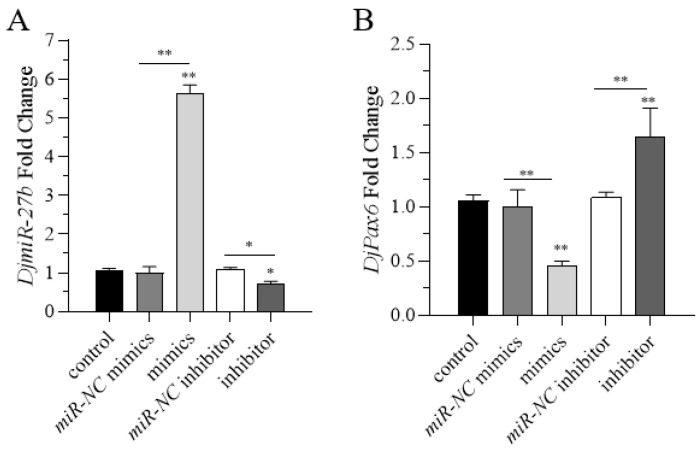
Expression level of *DjmiR-27b* and the target gene *DjPax6.* (**A**): Overexpression and interference efficiency of *DjmiR-27b*; (**B**): Expression level of the target gene *DjPax6* after overexpression and interference of *DjmiR-27b* (* *p* < 0.05; ** *p* < 0.01).

To explore the correlative expression between *DjmiR-27b* and its target gene *DjPax6*, overexpression and inhibition of *DjmiR-27b* were carried out with the *miR-NC* mimics group and the *miR-NC* inhibitor group as controls, and then qPCR was used to detect the expression levels of *DjmiR-27b* and *DjPax6*. As shown in Figure 4A, compared to the corresponding control, the overexpression group had significantly upregulated *DjmiR-27b*, while the *miR-NC* mimics group showed no significant change. The inhibition group had significantly downregulated *DjmiR-27b*, and the *miR-NC* inhibitor group had no significant change, indicating successful *DjmiR-27b* overexpression and inhibition. The expression level of *DjPax6* is shown in Figure 4B, *miR-NC* was used as a control. Compared to the control, the *DjmiR-27b* overexpression group had significantly reduced the expression level of *DjPax6*, while the *DjmiR-27b* inhibition group had significantly upregulated that of *DjPax6*. This indicates a negative correlation between the expression of *DjmiR-27* and *DjPax6* in planarians. When the expression level of *DjmiR-27* increases, the expression level of *DjPax6* decreases, while when the expression level of *DjmiR-27b* decreases, the expression level of *DjPax6* increases.

### 2.4. Targeting Analysis of DjmiR-27b and DjPax6

**Figure 5:**
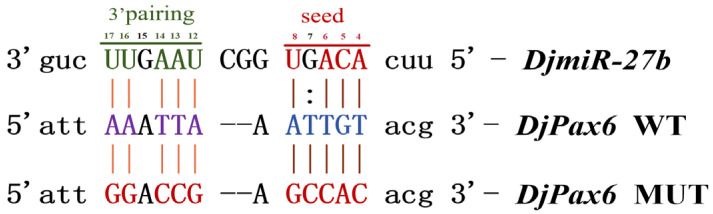
Binding site sequences and mutation sequences of target gene *DjPax6*.

**Figure 6:**
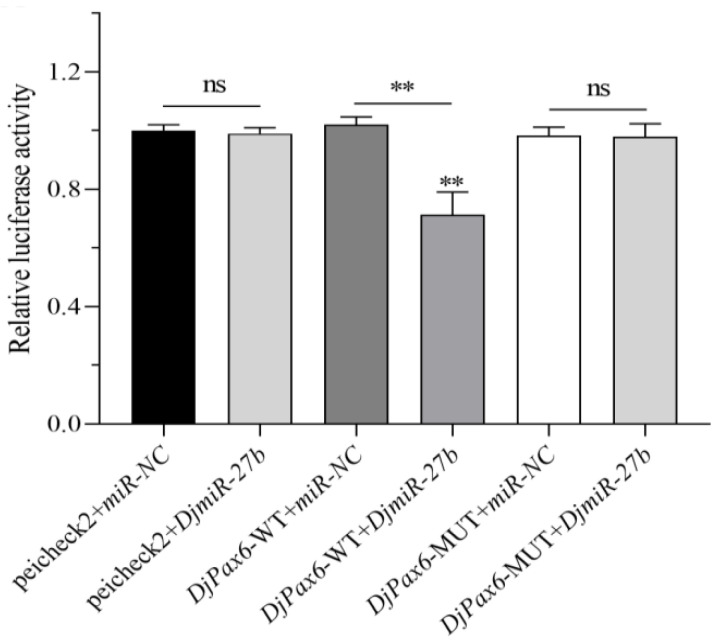
Double luciferase reporter gene experiment identifies the binding relationship between *DjmiR-27b* with target gene *DjPax6* (ns, not significant; ** *p* < 0.01).

**Figure A1:**
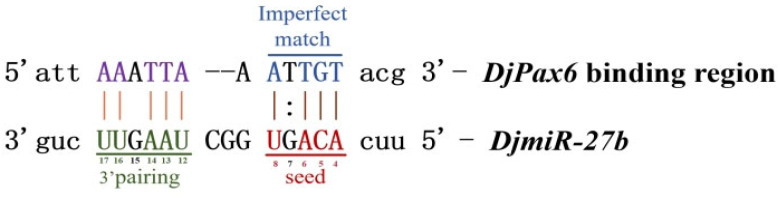
The binding sites of the binding region of target gene *DjPax6* to the seed sequences of *DjmiR-27b*.

**Figure A2:**
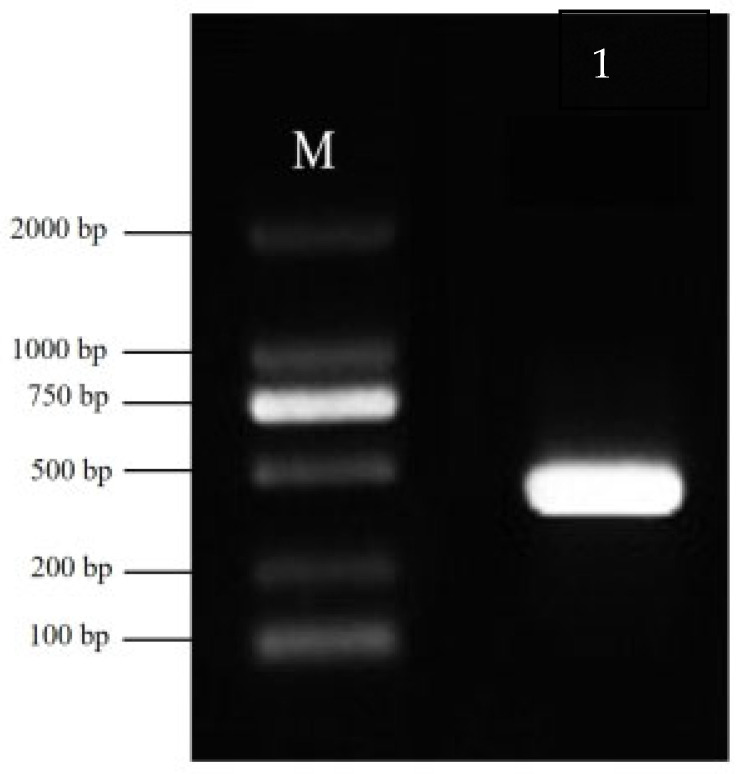
Cloning of the binding region of the planarian *DjPax6*. Notes M: DL 2000 DNA Marker; 1: DjPax6.

**Figure A3:**
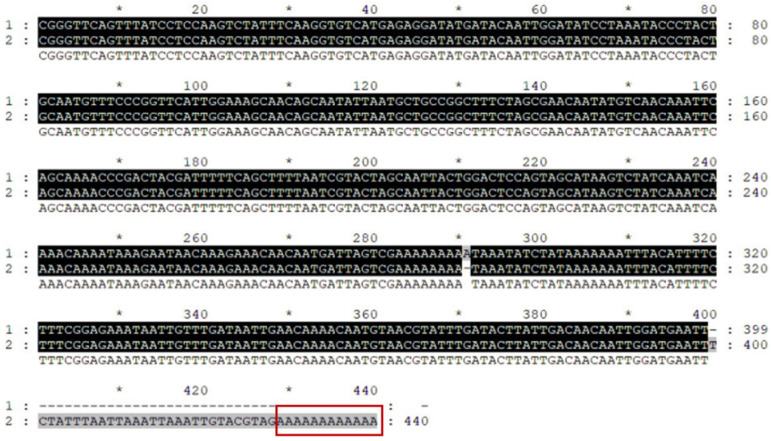
Alignment of transcriptome sequence and sequencing result for the binding region of *DjPax6* in planarians. Notes 1: Transcriptome sequence; 2: Sequencing sequence; The red box represents the poly A tail, and the * indicates a ten-base interval.

**Figure A4:**
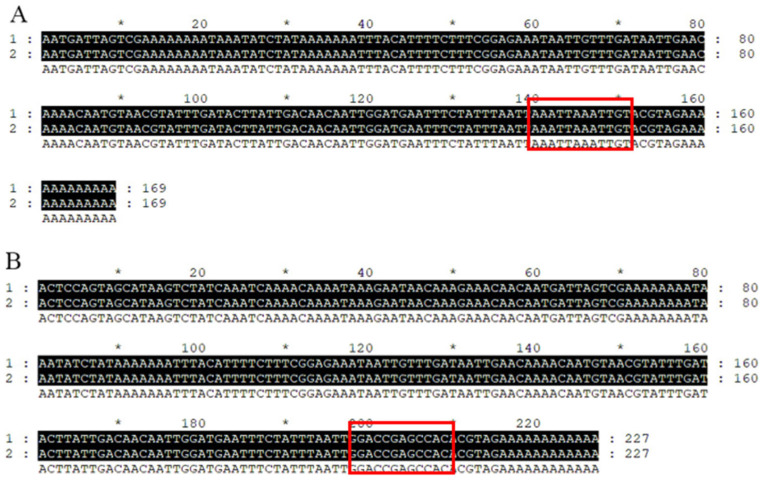
Alignment results of WT and MUT sequences of *DjPax6*. (**A**): *DjPax6* WT; (**B**): *DjPax6* MUT 1: Transcriptome sequence; 2: Sequencing sequence; The red boxes represent the binding site sequences of the wild and mutant types. and the * indicates a ten-base interval.

Using microinformatics analysis, it was found that the binding region of *DjPax6* and the seed sequence of *DjmiR-27b* have a binding energy of −2.67 (Appendix B Figure A1), and a binding energy less than −1 indicates good binding efficiency. To confirm the binding relationship between *DjmiR-27b* and *DjPax6*, the 3′UTR region of the target gene *DjPax6* binding to *DjmiR-27b* was amplified by PCR (Appendix B Figure A2) and cloned into the pUC19 vector. Sequencing results are shown in Appendix B, Figure A3. The binding sites of the *DjPax6* gene with *DjmiR-27b* were mutated based on the principle of exchanging A and G, T and C (Figure 5). Approximately 150–200 bp sequences flanking the wild-type (WT) and mutant (MUT) binding sites of *DjPax6* were designed (Appendix B Figure A4). The *XhoI*/*NotI*-digested psicheck2 vector was ligated with *DjPax6* WT or MUT fragments using T4 DNA ligase, thereby constructing the recombinant plasmids psicheck2+*DjPax6*-WT and psicheck2+*DjPax6*-MUT. Subsequently, psicheck2 and *miR-NC*, as well as psicheck2 and *DjmiR-27b* mimics, were transfected into 293T cells, respectively, for dual-luciferase assays. As shown in Figure 6, compared with the control group psicheck2+*miR-NC*, the luciferase activity of psicheck2+*DjmiR-27b* showed no significant change, indicating no binding site between psicheck2 and *DjmiR-27b*. To verify the binding relationship between *DjmiR-27b* and *DjPax6*, *DjmiR-27b* mimics and the psicheck2 recombinant plasmids containing the WT and MUT types of the target gene *DjPax6* were transfected into 293T cells, respectively, for dual-luciferase assays. The control groups were *DjPax6*-WT+*miR-NC* and *DjPax6*-MUT+*miR-NC*, and the experimental groups were *DjPax6*-WT+*DjmiR-27b* and *DjPax6*-MUT+*DjmiR-27b*. The results showed that compared with the control group, the dual-luciferase activity of the *DjPax6*-MUT+*DjmiR-27b* group showed no significant change, while that of the *DjPax6*-WT+*DjmiR-27b* group was significantly reduced, indicating that there is a binding site for *DjmiR-27b* in *DjPax6*-WT.

### 2.5. The Function of DjmiR-27b and DjPax6 Genes in Metformin-Regulated Planarian Eyespots Regeneration

**Figure 7:**
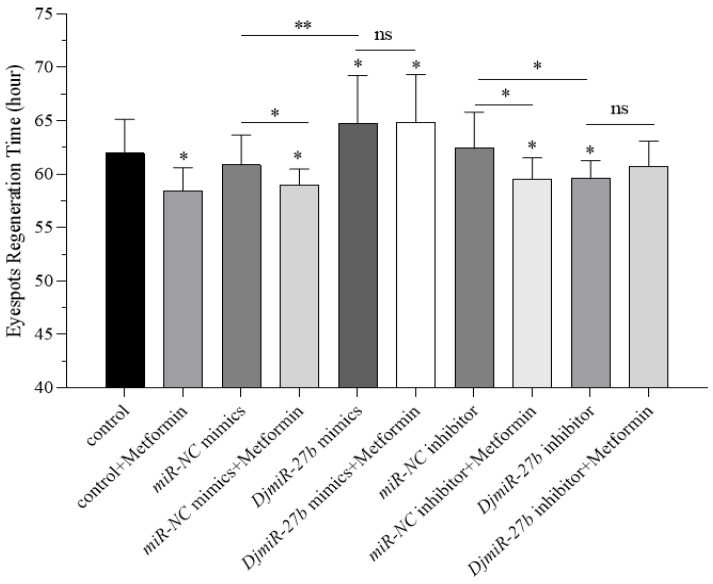
Eyespot regeneration time in planarians after overexpression and inhibition of *DjmiR-27b* (* *p* < 0.05; ** *p* < 0.01; ns, not significant).

To study the role of *DjmiR-27b* in metformin-regulated planarian eyespot regeneration, overexpression and inhibition of *DjmiR-27b* in planarians were conducted, with *miR-NC* mimics and *miR-NC* inhibitors as controls. After eyespot removal, planarians were cultured in clean water and 1 mM metformin, respectively, and the regeneration time was recorded. As shown in Figure 7, in clean water, the *DjmiR-27b*-overexpressed group had a significantly longer eyespot regeneration time than the control (*miR-NC* mimics), while the *DjmiR-27b*-inhibited group had a significantly shorter regeneration time than its control (*miR-NC* inhibitor). This indicates that *DjmiR-27b* overexpression suppresses eyespot regeneration, whereas its lower expression promotes regeneration. In metformin, the control, *miR-NC* mimics, and *miR-NC* inhibitor groups had significantly shorter eyespot regeneration times than those in clean water. However, in metformin, the eyespot regeneration times of the *DjmiR-27b*-overexpressed and inhibited groups (*DjmiR-27b* mimics+Metformin, *DjmiR-27b* inhibitor+Metformin) were similar to those of the clean water groups (*DjmiR-27b* mimics, *DjmiR-27b* inhibitor). Also, in 1 mM metformin and clean water, the *DjmiR-27b*-overexpressed groups had longer eyespot regeneration times than the *DjmiR-27b*-inhibited groups. This implies that after *DjmiR-27b* is overexpressed or inhibited, metformin can no longer promote eyespot regeneration. Based on the above results, it is hypothesized that metformin could regulate planarian eyespot regeneration by modulating the expression of *DjmiR-27b*.

**Figure A5:**
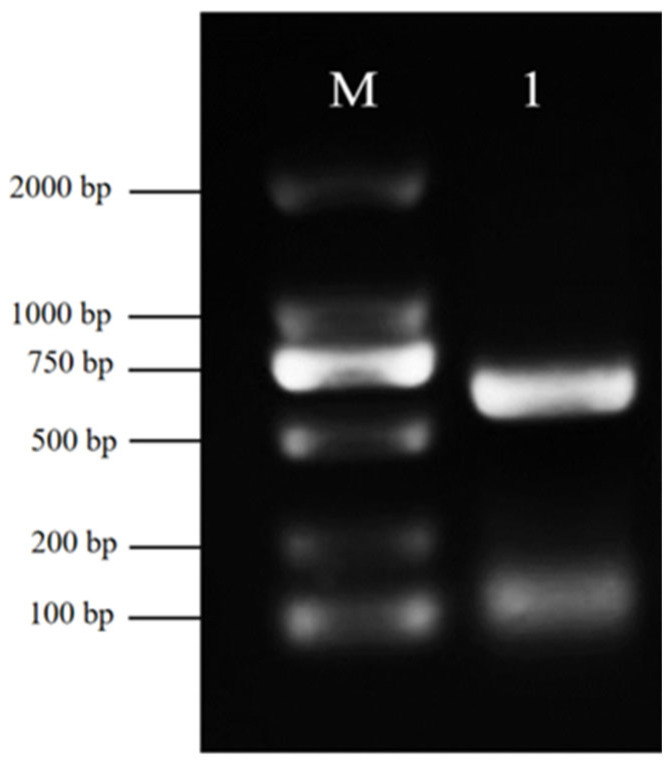
Cloning of the RNAi region of the planarian gene *DjPax6*. Notes M: DL 2000 DNA Marker; 1: *DjPax6*.

**Figure A6:**
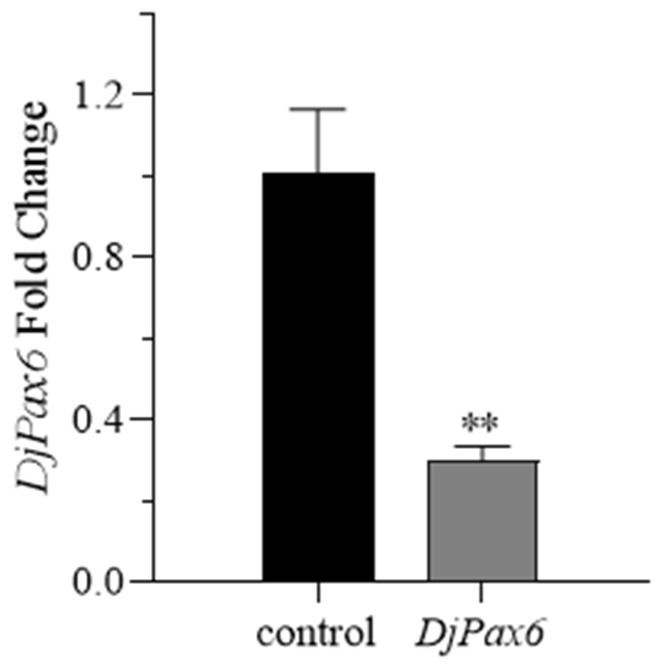
Detection of *DjPax6* interference efficiency (** *p* < 0.01).

To study the role of *DjPax6* in metformin-regulated planarian eyespot regeneration, a partial fragment of *DjPax6* (Appendix B Figure A5) was cloned into plasmid L4440, and the recombinant plasmid L4440-*DjPax6* was constructed. The bacterial cells induced by recombinant plasmid were mixed with nematode homogenate and fed to planarians to interfere with *DjPax6*. Subsequently, qPCR was employed to assess the interference efficiency of *DjPax6*. Results showed that the expression level of *DjPax6* was significantly reduced in the interference group, with an interference efficiency of about 63% (Appendix B, Figure A6).

**Figure 8:**
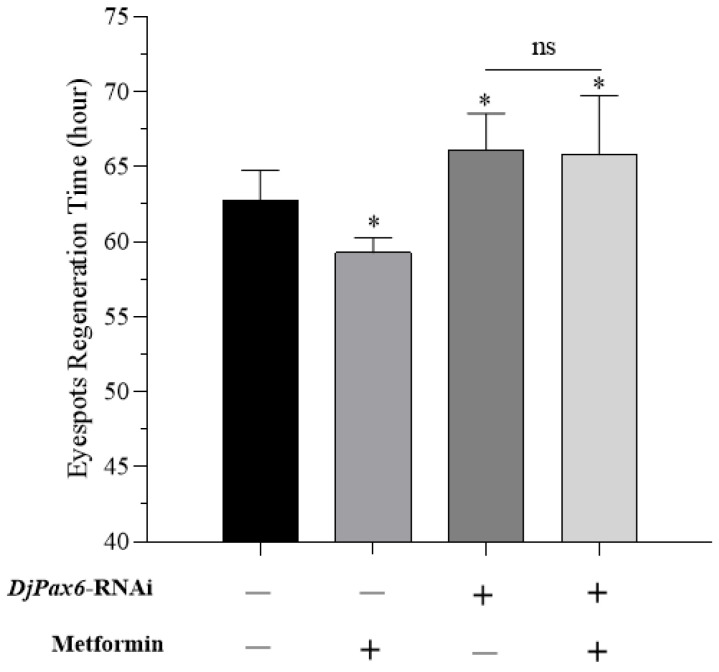
The regeneration time of eyespots in planarians after RNAi with *DjPax6* (* *p* < 0.05; ns, not significant).

After the eyespots of *DjPax6*-knockdown planarians were cut, they were cultured in clean water and 1 mM metformin, respectively, and the regeneration times were recorded. As shown in Figure 8, in non-knockdown planarians, the metformin-treated group exhibited significantly shorter regeneration times than the clean water group. In water-cultured planarians, the *DjPax6*-knockdown group showed significantly longer regeneration times than the non-knockdown group, indicating that *DjPax6* suppression inhibits eyespot regeneration. In the *DjPax6*-knockdown group, metformin treatment caused no significant difference in regeneration time compared to the clean water group, demonstrating that metformin fails to promote regeneration when *DjPax6* is knocked down. These results suggest that metformin regulates planarian eyespot regeneration by modulating *DjPax6* expression.

In summary, overexpression and inhibition experiments revealed that there is a negative regulatory relationship between the expression of *DjmiR-27b* and *DjPax6*. When *DjmiR-27b* is overexpressed or inhibited, metformin loses its ability to promote eyespot regeneration, showing that its effect depends on normal *DjmiR-27b* regulation. After *DjPax6* interference, metformin’s promoting effect on eyespot regeneration also disappears. Therefore, we speculate that metformin may inhibit the expression of *DjmiR-27b* to relieve its negative regulation on the target gene *DjPax6*, promote the expression of *DjPax6*, and ultimately regulate the regeneration of the eyespots of the planarians.

### 2.6. Distribution of DjmiR-27b and Its Target Gene DjPax6 in Planarians

**Figure 9:**
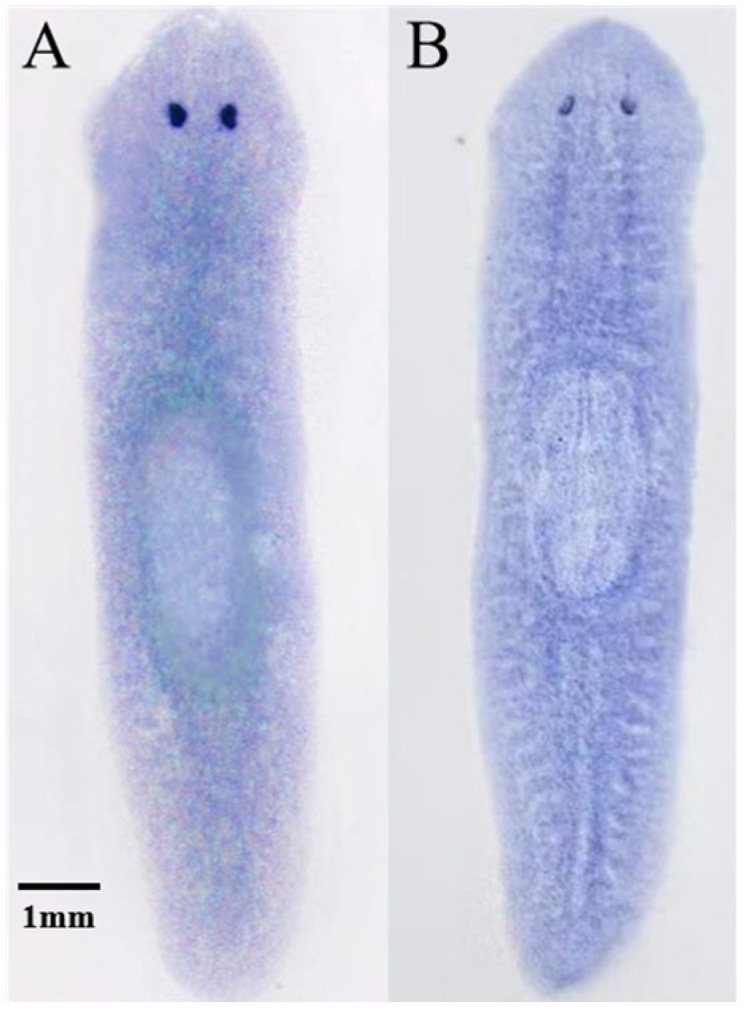
In situ hybridization detection of *DjmiR-27b* (**A**) and *DjPax6* (**B**) gene distribution in intact planarians.

In situ hybridization showed that *DjmiR-27b* and its target gene *DjPax6* are widely expressed in planarian tissues, including the head. *DjmiR-27b* expression is mainly around the pharynx, with slightly weaker expression in the pharynx. *DjPax6* is particularly concentrated at the eyespot nerve location (Figure 9). The results of the preliminary functional experiments are consistent with the spatial distribution patterns, indicating that in the early stage of head regeneration, *DjmiR-27b* may participate in regulating eyespot regeneration by negatively regulating *DjPax6* activity.

## 3. Discussion

In recent years, research has found that metformin is not only a drug for the treatment of type 2 diabetes but is also associated with the regeneration of tissues and organs [1,11,12,54]. Studies have shown that 50 μM metformin can enhance autophagic flux and improve the regeneration of the epicardium, endocardium, and vascular endothelium in zebrafish, promote autophagic flux activation, accelerating regeneration of epicardial, endocardial, and vascular endothelial tissues in zebrafish [12]. In vitro experiments with C57BL/6 mice, treatment with 100 µM metformin increases phosphorylated AMPK levels, restoring the differentiation capacity of senescent oligodendrocyte precursor cells and thereby improving central nervous system CNS remyelination [11]. Lei et al. found that 0.1 mM metformin can promote the proliferation of human umbilical cord mesenchymal stem cells and enhance their osteogenesis and angiogenesis [55]. These studies indicate that metformin has the effect of promoting regeneration, and the concentration of promoting regeneration varies in different tissues and organs. Similarly, the planarian eyespot regeneration experiments in this study revealed that 0.01 mM, 0.1 mM, and 1 mM metformin significantly promoted eyespot regeneration, whereas 10 mM metformin had the opposite effect. In this experiment, the regeneration time of eyespots after treatment with metformin in regenerating planarians was different from Zhao’s results [52]. This may be due to the seasonal rhythm of planarians’ regeneration. Although the constant temperature of the incubator eliminates the influence of temperature changes as an environmental factor on planarians, planarians still have endogenous circadian rhythms and rhythmic responses to non-temperature environmental factors. The regeneration of planarians has a certain seasonal rhythm [56,57,58]. Therefore, in our experiment, we indeed found that there were differences in the regeneration rate of planarian eyespots among batches from different seasons. However, for the same batch of planarians, the regeneration rate of the eyespots of planarians cultured in low concentrations of metformin is indeed significantly faster than that of those cultured in clean water, indicating that metformin can accelerate the regeneration of planarians.

MiRNAs are involved in various physiological activities such as cell proliferation, differentiation, migration, apoptosis, and tissue metabolism in organisms [30]. Studies have shown that metformin exerts its biological effects by regulating the expression of various miRNAs. Metformin could increase the expression of *miR-497a-5p* in mouse eyes and alleviate the severity of their retinal lesions [59]. Metformin could delay cell aging, cellular senescence in human dental pulp stem cells (DPSCs) by downregulating *miR-34a-3p* in human dental pulp stem cells [60] and reduce lipid accumulation in high glucose-induced HepG2 cells by downregulating *miR-33b* expression [61]. Research in planarians has found that *miR-8b* serves as a crucial regulator involved in brain and eyespot regeneration in *D. japonica* [34]. Loss of the *miR-124* family could lead to a significant reduction in the regenerative capacity of the brain and visual system in *Schmidtea mediterranea* [62]. Therefore, we speculate that metformin might affect the regeneration of *D. japonica* by regulating the expression of miRNA. Through the study of the effect of metformin on the expression of *DjmiR-27b* and *DjPax6* in the regenerating planarians, we found that low concentrations of metformin significantly inhibited the expression of *DjmiR-27b* and promoted the expression of *DjPax6*. There is a negative regulatory relationship between *DjmiR-27b* and *DjPax6*. The dual-luciferase reporter assay confirmed that *DjmiR-27b* directly targets the binding site of the 3′UTR of the *DjPax6* gene, so it is speculated that metformin may regulate planarian regeneration by regulating the expression level of the target gene *DjPax6* through *DjmiR-27b*.

*Pax6* is a key regulator of eye development in numerous organisms, which is involved in eye development in *Drosophila*, *Capitella teleta*, and mammals [63,64,65]. *Xenopus tropicalis Pax6* is expressed in various eye tissues, including the neural retina layers, retinal neuroepithelium, optic disc, corneal epithelium, iris, and ciliary body [66,67]. Abnormal expression of *Pax6* could lead to abnormal eye development, resulting in microphthalmia microphthalmos in mice [68,69] and anophthalmia anophthalmos in fruit flies [70]. The research of Pineda D et al. found that *Pax6* knockdown in planarians did not disrupt normal eyespot development, leading to the conclusion that *Pax6* has no role in planarian eyespot regeneration [71]. However, our study reveals that although *Pax6* knockdown did not cause morphological eyespot regeneration defects, it significantly prolonged the eyespot regeneration time, indicating the involvement of *Pax6* in planarian eyespot regeneration.

Studies have shown that *miR-27b* is involved in tissue regeneration, metabolism, and other biological processes by regulating its target genes or related signaling pathways. Studies on human microvascular endothelial cells (HMEC)-1 have shown that downregulation of *miR-27b* can activate the PI3K/AKT signaling pathway and promote angiogenesis [72]. By targeting the regulation of *HOXC6* expression, *miR-27b* could inhibit epithelial–mesenchymal transition in human retinal pigment epithelial cells, reducing retinal fibrosis fibrotic retinopathy in patients [73]. In our work, overexpression of *DjmiR-27b* and inhibition of its target gene *DjPax6* both significantly prolonged the regeneration time of planarian eyespots, whereas knockdown of *DjmiR-27b* significantly shortened the regeneration time. This suggests that overexpression of *DjmiR-27b* downregulates its target gene *DjPax6*, thereby inhibiting eyespot regeneration in planarians. Conversely, inhibition of *DjmiR-27b* upregulates *DjPax6* expression and promotes eyespot regeneration. Combining the regulatory relationship between miRNA and target genes, it is speculated that metformin may inhibit the expression of *DjmiR-27b*, thereby promoting the expression of the target gene *DjPax6* and regulating the process of regeneration in regenerating planarians. Previous studies have demonstrated that *miR-27b* could promote muscle regeneration in mice after injury by targeting the target gene *MDFI*, and the expression of type II collagen during the differentiation of rat articular chondrocytes by regulating the expression of the target gene *Pparγ2* [74,75]. Therefore, as an important regulatory factor, *miR-27b* can regulate cell proliferation, differentiation, and metabolism by modulating different target genes, thereby affecting tissue development and repair.

In situ hybridization has been utilized to detect the spatial expression patterns of genes in organisms. So far, only a few studies have been reported for the expression localization of miRNAs in planarians [34,62]. *DjmiR-124* and *DjmiR-8b* are involved in the regeneration of the eye or brain in planarians. *DjmiR-8b* is specifically expressed in the head and eyespots of planarians [34], and *DjmiR-124* is predominantly localized to the cephalic ganglia, ventral nerve cords, and visual system [12]. The morphogenesis of eyespots is crucial for head regeneration in planarians. Results of in situ hybridization showed that *DjmiR-27b* and its target gene *DjPax6* are widely expressed in planarian head tissue. *DjmiR-27b* likely regulates eyespot regeneration by targeting *DjPax6* in the early stage of head regeneration.

The results of this study reveal the regulatory effects of metformin on *DjmiR-27b* and *DjPax6,* as well as their targeting relationship, indicating that metformin may modulate planarian eyespot regeneration by regulating *DjPax6* expression via *DjmiR-27b*. This research sheds light on the potential molecular mechanisms by which metformin regulates planarian regeneration, providing a theoretical basis for studying metformin in regenerative medicine. However, the regeneration of planarian eyespots involves complex molecular regulatory mechanisms. As a drug, metformin does not regulate planarian regeneration in a single-targeted manner. *DjmiR-27b* is just one of the many molecules involved in regulating regeneration. Further studies beyond the *DjmiR-27b*/*Pax6* axis will enhance our understanding of the molecular mechanisms by which metformin regulates planarian regeneration.

## 4. Materials and Methods

### 4.1. Experimental Animal

The planarian used in this study was an asexual strain *Dugesia ZB-1* kept in our laboratory [76]. The animals were cultured in *Montjuïc water* (1.6 mM NaCl, 1 mM CaCl_2_, 1 mM MgSO_4_, 0.1 mM MgCl_2_, 0.1 mM KCl, and 1.2 mM NaHCO_3_) (Beijing Solarbio Science & Technology Co., Ltd., Beijing, China) in an incubator at 20 °C. Planarians were fed with nematode homogenate once a week. Before the experiments, planarians were subjected to a week-long period of starvation.

### 4.2. Experimental Material

*E. coli* HT115 and L4440 vectors were preserved by our laboratory. *E. coli* DH5α competent cells were purchased from Shanghai Angyu Biotechnology Co., Ltd. (Shanghai, China). The restriction enzymes *XhoI* and *PstI*, along with T4 DNA ligase, were purchased from Takara Bio Inc. (Kusatsu, Shiga, Japan). Revertra Ace qPCR RT Kit was obtained from Toyobo Co., Ltd. (Osaka, Japan). Dual-luciferase reporter vector psiCHECK2 and 293T cells were provided by Biotech Primer Co., Ltd. (Beijing, China). LipoFiter Transfection Reagent was purchased from Shanghai Hanbiotechnology Co., Ltd. (Shanghai, China). *DjmiR-27b* mimics, *DjmiR-27b* inhibitor, and *DjmiR-27b*-specific probe were synthesized by Sangon Biotech (Shanghai) Co., Ltd. (China). All primers were commercially synthesized by Beijing Genomics Institute (BGI), and their sequences are listed in Appendix A, Table A1, Table A2, Table A3, Table A4, Table A5, Table A6, Table A7 and Table A8. In addition, 4% paraformaldehyde (PFA) was purchased from Wuhan Service Biotechnology Co., Ltd. (Wuhan, China). T7 RNA Polymerase and RNase Inhibitor were purchased from Beijing GenStar Biotechnology Co., Ltd. (Beijing, China). DIG, DIG antibody, and NBT/BCIP were purchased from F. Hoffmann-La Roche Co., Ltd. (Basel, Switzerland). Maleic acid, sodium heparin, and Tween 20 were obtained from Sigma-Aldrich Co. LLC (St. Louis, MO, USA). In addition, 20 × SSC were purchased from Shanghai Macklin Biochemical Co., Ltd. (Shanghai, China). Yeast RNA and 50×Denhardt’s solution were obtained from Beijing Solarbio Science & Technology Co., Ltd. (Beijing, China).

### 4.3. Determination of DjmiR-27b and DjPax6 Gene as Research Subjects

Two databases generated from our previous work were analyzed: the miRNA database of the regenerating planarians [53] and the transcriptome database of metformin-treated regenerating planarians [52]. Downregulated miRNAs were selected from the miRNA database, and upregulated genes were identified from the transcriptome database. Target genes for the selected miRNAs were predicted using TargetScan (https://www.targetscan.org/vert_72/) (accessed on 19 July 2023). Consequently, *DjmiR-27b* and its target gene *DjPax6* were chosen as the research targets.

### 4.4. Planarian Eyespot Regeneration Experiment

A total of 50 planarians with a body length of approximately 0.8–1 cm were selected. Eyespots were surgically removed posterior to the auricles under SZ650 stereomicroscopy (Chongqing Aote Optical Instruments Co., Ltd., Chongqing, China). The regenerating planarians were divided into five groups, with ten individuals in each group. Four experimental groups were placed in metformin solutions of 0.01 mM, 0.1 mM, 1 mM, and 10 mM, respectively, while the control group was placed in clear water. Solutions were replaced daily to ensure consistent drug concentration. Eyespot regeneration was observed every 12 h within the first 48 h, then hourly after 48 h. Photos were taken at 0 h, 12 h, 24 h, 48 h, 72 h, and 96 h. When two distinct and symmetrical black eyespots could be clearly observed under the stereomicroscope, the corresponding time was recorded as the eyespots’ regeneration time. The experiment was repeated three times.

### 4.5. RT-qPCR

Total RNA was extracted from planarians using Trizol reagent (Thermo Fisher Scientific Inc., Waltham, MA, USA). Reverse transcription was performed with Oligo (dT)_15_ primers (Beijing Genomics Institute, Shenzhen, Guangdong, China) using ReverTra Ace qPCR RT Kit (Toyobo Co., Ltd., Osaka, Japan) according to the manufacturer’s protocol. Quantitative PCR (qPCR) reaction mixture (10 µL) contained 1 µL cDNA of 10 ng/µL, 0.4 µL 10 µM forward primer, 0.4 µL 10 µM reverse primer, 5 µL TB Green Premix Ex Taq II (2×), and 3.2 µL ddH_2_O. Amplification was performed on a Light Cycler 480 system (Roche Diagnostics, Basel, Switzerland) with the following protocol: initial denaturation at 95 °C for 30 s, followed by 40 cycles of 95 °C for 5 s and 60 °C for 30 s. The experiment was repeated three times. *DjActin* gene was used as an endogenous reference, and the expression levels of genes were calculated using the 2^−ΔΔCt^ method.

### 4.6. Dual-Luciferase Reporter Assay

The binding sites and energy between miRNA and target genes were predicted using the Bioinformatics Platform (http://www.bioinformatics.com.cn) (accessed on 19 July 2023). Site-directed mutagenesis was performed on the miRNA-binding regions with A→G and T→C substitutions. About 200 bp sequences with *XhoI* and *NotI* restriction sites flanking the wild-type (WT) and mutant (MUT) binding sites of *DjPax6* were synthesized by Hunan Pulettech Biotechnology Co., Ltd. (Changsha, Hunan, China). These fragments were cloned into the psiCHECK-2 dual-luciferase reporter vector using *XhoI/NotI* digestion and T4 DNA Ligase, then transformed into DH5α cells. Positive clones were sequenced by Suzhou Genewiz Biotechnology Co., Ltd. The validated plasmids were transfected into 293T cells, incubated for 24 h at 37 °C with 5% CO_2_, and lysed with Passive Lysis Buffer. Firefly and Renilla luciferase activities were measured sequentially using the Lux-T020 Detector (Guangdong Biolight Medical Equipment Co., Ltd., Zhuhai, China). The instrument settings were: 2 s interval, 10 s measurement time, and 40–100 µL sample volume. Firefly luciferase activity was measured first, followed by Renilla luciferase activity as an internal reference.

### 4.7. DjmiR-27b Overexpression and Inhibition Experiments

The experiments of overexpression and knockdown of *DjmiR-27b* in planarians were performed through the feeding method. Each food homogenate contained 15 µL nematode homogenate mixed with 5 µL 2% low-melting-point agarose (Biosharp, Hefei, Anhui, China). A total of 30 planarians with a body length of 0.6–0.8 cm were selected for each experimental group. The overexpression group was fed with 8 µL of *DjmiR-27b* mimics mixed with 20 µL of food homogenate. The knockdown group was fed with 8 µL of *DjmiR-27b* inhibitor mixed with 20 µL of food homogenate. The negative control group was fed with 8 µL *DjmiR-27b* negative control (NC) mixed with 20 µL food homogenate, and the blank control group was fed with 8 µL RNase-free water mixed with 20 µL food homogenate. Planarians were fed once a day for 10 consecutive days. Total RNAs were then extracted from the planarians, and RT-qPCR was performed to assess the efficiency of *DjmiR-27b* overexpression and knockdown. The experiment was repeated three times.

### 4.8. RNA Interference (RNAi) of DjPax6 Gene

Total RNA was extracted from planarians and reverse-transcribed into cDNA. The amplified *DjPax6* gene fragment and plasmid L4440 were digested with *XhoI* and *PstI* and then ligated using T4 DNA Ligase (Takara Bio Inc. Kusatsu, Shiga, Japan). Sequences of *DjPax6* interference primers are listed in Appendix A, Table A6. L4440-*DjPax6* recombinant plasmid and empty L4440 plasmid were, respectively, transformed into HT115 competent cells and induced with 1 mmol/L IPTG (Beyotime Biotechnology Co., Ltd., Shanghai, China). After induction, the bacterial cells were mixed with nematode homogenate at a 1:2 weight ratio and fed to planarians for five consecutive days. A total of 200 planarians were evenly divided into two groups. The RNAi-treated group was fed with a mixture of bacteria expressing the recombinant plasmid L4440-*DjPax6* and nematode homogenate, while those fed with mixture of bacteria expressing the empty L4440 plasmid and nematode homogenate served as control group. Total RNAs were extracted from the planarians and reverse transcribed into cDNAs on the next day after the final feeding. RNAi efficiency was assessed by RT-qPCR. After the amputation of eyespots, 100 planarians of each group were cultured in fresh water and metformin solution, respectively. A total of 200 planarians were divided into four groups: (1) freshwater-cultured control planarians, (2) freshwater-cultured RNAi-treated planarians, (3) metformin-treated non-RNAi planarians, and (4) metformin-treated RNAi-treated planarians. The regeneration time of planarian eyespots was recorded.

### 4.9. In Situ Hybridization

Ten planarians with a body length of approximately 3–5 mm were selected for the experiment. DIG-labeled antisense probes for *DjmiR-27b* and *Djpax6* were synthesized by BGI Genomics Co., Ltd. (Shenzhen, Guangdong, China). Planarians were fixed with 4% PFA (Wuhan Service Biotechnology Co., Ltd., China). at room temperature for 1 h, followed by sequential dehydration with 50% and 100% methanol, and then bleached under strong light with 6% Bleach Solution overnight. Rehydration was performed with 100% and 50% methanol. Subsequently, the samples were washed for 10 min with a solution of PBST (Beijing Solarbio Science & Technology Co., Ltd., Beijing, China) and PreHyb mixed in a 1:1 ratio, pre-hybridized for 1.5 h in pre-hybridization solution, and then hybridized for more than 16 h in hybridization solution containing a final concentration of 0.1 to 1 ng/µL of the probe, with temperature maintained at 56 °C. Planarians were blocked with Blocking Solution at room temperature for 2 h and then incubated overnight at 4 °C in Blocking Solution containing anti-DIG antibody (F. Hoffmann-La Roche Co., Ltd., Basel, Switzerland). They were washed with Alkaline Phosphatase Buffer (F. Hoffmann-La Roche Co., Ltd., Basel, Switzerland). and developed in the dark on a shaker with a solution mixed in a 1:50 ratio of NBT/BCIP and AP Buffer until they turned purple-red. Samples were imaged by a stereomicroscope (Nikon SMZ 1500, Tokyo, Japan).

### 4.10. Bioinformatics Analysis

Nucleotide sequences were translated into protein sequences using the ExPASy Translate tool (https://web.expasy.org/translate/) (accessed on 19 July 2023). Multiple sequence alignment of sequencing results was performed using GeneDoc software 2.7. Statistical analyses were conducted with SPSS 26.0. Group comparisons were analyzed by one-way ANOVA, with significance levels set at *p* < 0.05 for statistical significance and *p* < 0.01 for extremely significant differences. Data analysis and plotting were carried out using GraphPad Prism 5.

## 5. Conclusions

This study preliminarily explores the molecular mechanism by which metformin regulates planarian regeneration through miRNAs, uncovering a targeting relationship between *DjmiR-27b* and *DjPax6*. Our findings demonstrate that metformin influences planarian eyespot regeneration by modulating *DjmiR-27b* and *DjPax6*. The results of this study will provide a theoretical basis for the application of metformin in the field of regeneration.

## Data Availability

Data are contained within the article.

## Appendix A

**Table A1:** The partial target genes of *DjmiR-27b*.

miRNA Name	Target Gene Name
*DjmiR-27b*	*Pax6*, *AFG3L2*, *glt1*, *ITSN1*, *ERF1*, *Eml1*, *St13*, *UBE2L3*, *SLC2A4*, *Vps39*, *Pax5*

**Table A2:** The reverse transcription primer of miRNA.

Primer Name	Sequence (5′-3′)
*DjmiR-27b*-RT	GTCGTATCCAGTGCGTGTCGTGGAGTCGGCAATTGCACTGGATACGACCAGAAC

**Table A3:** The qPCR primers of miRNA.

Primer Name	Sequence (5′-3′)
*β-DjActin*-F	ACCAGCAGATTCCATACCCA
*β-DjActin*-R	TTATGTTACGTTGCCCTCGA
*DjmiR-27b*-F	CAGTGCGTGTCGTGGAGT
*DjmiR-27b*-R	GGGGTTCACAGTGGCTAA

**Table A4:** The qPCR primers of miRNA target gene.

Primer Name	Sequence (5′-3′)
*β-DjActin*-F	ACCAGCAGATTCCATACCCA
*β-DjActin*-R	TTATGTTACGTTGCCCTCGA
*DjPax6*-F	ACTATCGGTTGTTGATTGGAC
*DjPax6*-R	ACTGCGGTGCTTATGGTG

**Table A5:** Sequences of miRNA mimics and inhibitor.

Primer Name	Forward Sequence (5′-3′)	Reverse Sequence (5′-3′)
*DjmiR-27b* mimics	UUCACAGUGGCUAAGUUCUG	CAGAACUUAGCCACUGUGAA
*DjmiR-27b* inhibitor		CAGAACUUAGCCACUGUGAA

**Table A6:** The primer sequences for RNAi.

Primer Name	Sequence (5′-3′)
*DjPax6*-F	CCGCTCGAGACTGCGGTGCTTATGGTG
*DjPax6*-R	AACTGCAGCGGCTGATAATGAAGGAGTTT

**Table A7:** The primers of 3′RACE.

Primer Name	Sequence (5′-3′)
3′RACE outer primer	TACCGTCGTTCCACTAGTGATTT
3′RACE inner primer	CGCGGATCCTCCACTAGTGATTTCACTATAGG
3′RACE *DjPax6* outer primer	ACCAAATCTTTCGCAATCTT
3′RACE *DjPax6* inner primer	CGGGTTCAGTTTATCCTCC

**Table A8:** The PCR primers of in situ hybridization.

Primer Name	Sequence (5′-3′)
ISH-*DjPax6*-F	CCGCTCGAGACTGCGGTGCTTATGGTG
ISH- *DjPax6*-R	GATCACTAATACGACTCACTATAGGGCGGCTGATAATGAAGGAGTTT
