# Correction: Yang et al. Molecular Mechanism of Metformin Regulating the Regeneration of Planarian *Dugesia japonica* Through *miR-27b*. *Int. J. Mol. Sci.* 2025, *26*, 7092

**DOI:** 10.3390/ijms27198916

**Published:** 2026-10-08

**Authors:** Kexin Yang, Minmin Feng, Chunmei Zhang, Zelong Zhao, Dandan Yin, Linxia Song, Zhenbiao Xu

**Affiliations:** School of Life Sciences and Medicine, Shandong University of Technology, Zibo 255000, China; 19811718781@163.com (K.Y.); minamin1997@126.com (M.F.); 18632842808@163.com (C.Z.); zelong0222@gmail.com (Z.Z.); yindandansdut@163.com (D.Y.); slxch@163.com (L.S.)

In the original publication [1], Reference 10, “Cejuela, M.; Martin-Castillo, B.; Menendez, J.A.; Pernas, S. Metformin and Breast Cancer: Where Are We Now? *Int. J. Mol. Sci.* **2022**, *23*, 2705”, has subsequently been retracted. To uphold the highest standards of academic integrity and ensure the accuracy of the scientific record, the authors decided to remove reference 10.

A correction has been made to Section 1. Introduction: “In addition, it has shown the ability to inhibit the growth and metastasis of various types of tumor cells, including breast cancer, liver cancer, and gastric cancer, breast, hepatic, and gastric carcinomas [9].”

Consequently, all subsequent references have been renumbered, and the in-text citation numbers have been updated accordingly. The authors state that the scientific conclusions are unaffected. This correction was approved by the Academic Editor. The original publication has also been updated.
